# Context and Multi-Features-Based Vulnerability Detection: A Vulnerability Detection Frame Based on Context Slicing and Multi-Features

**DOI:** 10.3390/s24051351

**Published:** 2024-02-20

**Authors:** Yulin Zhang, Yong Hu, Xiao Chen

**Affiliations:** School of Cyber Science and Engineering, Sichuan University, Chengdu 610207, China; 2021226245051@stu.scu.edu.cn (Y.Z.); chenxiao2@stu.scu.edu.cn (X.C.)

**Keywords:** vulnerability detection, graph neural network, context slicing, multi-features

## Abstract

With the increasing use of open-source libraries and secondary development, software projects face security vulnerabilities. Existing studies on source code vulnerability detection rely on natural language processing techniques, but they overlook the intricate dependencies in programming languages. To address this, we propose a framework called Context and Multi-Features-based Vulnerability Detection (CMFVD). CMFVD integrates source code graphs and textual sequences, using a novel slicing method called Context Slicing to capture contextual information. The framework combines graph convolutional networks (GCNs) and bidirectional gated recurrent units (BGRUs) with attention mechanisms to extract local semantic and syntactic information. Experimental results on Software Assurance Reference Datasets (SARDs) demonstrate CMFVD’s effectiveness, achieving the highest F1-score of 0.986 and outperforming other models. CMFVD offers a promising approach to identifying and rectifying security flaws in large-scale codebases.

## 1. Introduction

According to the annual report released by Skybox Security in 2022 [1], a total of 20,175 CVE vulnerabilities were disclosed in 2021, marking a 10% increase from the previous year. This represents a new pinnacle in the rate of vulnerability growth since 2018, and it is the first instance where the disclosure of vulnerabilities has exceeded the threshold of 20,000. The report underscores that the intensification of the demand for remote home offices has led to the widespread integration of software facilities across various industries, propelling the processes of societal electronification and informatization. However, this has concurrently broadened the attack surface of cybersecurity threats, resulting in an unprecedented number of security incidents.

The report particularly emphasizes that, beyond traditional IT security concerns, security issues in the Operational Technology (OT) domain are more disconcerting: a plethora of OT devices, especially Internet of Things (IoT) devices, are extensively employed in various enterprises. Due to the predominant deployment of IoT devices within enterprise intranets or their indirect connection to the internet, OT systems generally lack adequate security measures [2,3]. Nevertheless, with the continuous deepening of societal informatization, many IoT devices are currently accessing the internet through wired or wireless means. Siemens disclosed 518 OT-related vulnerabilities in 2021, and Hitachi also revealed 73 OT vulnerabilities. Undoubtedly, it can be stated that current OT systems have deteriorated into impending security time bombs.

IoT devices, as fundamental components of intelligent systems, extensively rely on various sensors to gather environmental data. The underlying code for these sensors is of-ten implemented using embedded programming languages such as C/C++, enabling direct hardware control and efficient performance. However, this implementation approach poses certain potential risks in terms of security. The inherent characteristics of the C/C++ languages make them susceptible to memory safety issues like buffer overflows, potentially leading to system vulnerabilities and security threats. Given these considerations, it becomes crucial and necessary to conduct automated vulnerability detection for the underlying code of sensors. By ensuring the security of the sensor code, the overall security of IoT devices can be effectively enhanced, thereby reducing the security risks faced by the entire IoT system.

At the same time, some open-source projects which are widely used as third-party libraries can contaminate the software supply chain, resulting in more profound and serious impacts, if vulnerabilities are caused by the open-source projects. The Log4shell [4] incident stands out as one of the most severe supply-chain-poisoning security events in recent years.

The aforementioned situation imparts practical significance to source code vulnerability detection, which has gradually become a prominent research domain in recent years.

The scrutiny of source code security is an indispensable and integral component of software development. In the early stages, manual code review constituted the primary method for security assessment. However, manual code inspection is time-consuming and tedious, and its efficacy heavily relies on individual expertise. With the evolution of the software industry, conducting manual reviews on a large scale has become impractical. Given the increasingly complex vulnerability patterns and the difficulty in detecting concealed triggering paths, relying solely on the results of multiple experts for manual review in a particular project would exhaust more human resources.

This has led researchers to believe that automation is the key to addressing the detection of vulnerabilities in complex, large-scale software source code. Early research employed detection methods based on code metrics [5,6,7]. Code metrics comprise a set of indicators that can represent information about the source code in a certain dimension, such as lines reflecting code complexity, McCabe, churn, and inheritance depth. Using these metrics, simple machine learning methods were employed to detect vulnerabilities in the source code. However, human involvement is still required in the design and extraction of code metrics. Moreover, this research approach does not directly analyze the source code itself; instead, it attempts to indirectly extract relevant metrics to obtain semantic information about the source code. These features fail to grasp information at the semantic level of the source code, thus yielding limited effectiveness.

In recent years, the remarkable advancements in natural language processing for text tasks have inspired researchers to adopt data-driven research approaches. Hindle et al. [8] highlighted the substantial similarities between programming language and natural language, characterized by a considerable degree of redundancy and predictable features. By employing various representation methods to transform source code into vector form, researchers leverage deep neural networks to autonomously learn patterns of vulnerabilities. These representation methods have evolved from simply treating source code as natural language text to transforming it into different graph structures, which are required for different deep-learning models to extract features.

We observe that the currently prevalent methods for feature extraction, particularly slicing, suffer from issues such as indistinct vulnerability features and the omission of crucial statements. Consequently, we propose a novel approach named “context slicing”. Additionally, we have devised an innovative vulnerability detection framework that integrates graph structure information with sequential information. Initially, we extract the Program Dependency Graph (PDG) from the source code and apply contextual slicing on this PDG to obtain cs-SDG (context slicing sub-PDG). To capture global semantic information, we use Graph Neural Networks (GNNs) to process the graph structure representation of the source code. Simultaneously, we utilize Sequence Neural Networks to analyze the source code text in cs-SDG, extracting local syntax information. Experimental results demonstrate that, in comparison to existing models, the proposed approach we proposed performs better in vulnerability detection.

This paper presents a new method of slicing and network architecture that offers the following contributions:

1. We introduce graph neural networks into the realm of source code vulnerability detection. Despite some current research no longer directly extracting features from the source code text sequences, these research methods still utilize sequence neural networks to extract features, necessitating the intermediate representation of graph structures to be converted back into sequence structures. In contrast, graph neural networks can directly assimilate the graph structure of the intermediate representation of the source code, mitigating the semantic loss incurred during the conversion process.
2. We introduce a novel code slicing technique named “Context Slicing”. Through data dependency and control dependency, context slicing can extract code lines highly relevant to the semantics of the slicing points, thereby reducing noise during training and enhancing the effective information density of the samples. Context slicing can split the complex and extensive PDG into logically, closely related and finer-grained semantic units, reducing the computational load required during the training process. Experimental results indicate that, in comparison to traditional methods, context slicing encompasses richer semantic information and vulnerability patterns, leading to an improvement in the detection performance of the model.
3. We propose a network architecture that seamlessly integrates global semantic features and local syntactic features. The fusion of graph and sequence features empowers the model to discern vulnerability patterns from both a holistic and intricate perspective. These dual perspectives are synergized to augment the expressive power of the extracted features. Experimental evaluations on five categories related to memory from the SARD dataset yielded a remarkable average F1-score of 0.950. This represents a significant advancement over contemporary models solely reliant on graph structures, affirming the robust efficacy of our proposed vulnerability detection framework.

The remaining sections of this paper will be organized as follows: Section 2 pro-vides a detailed introduction to common vulnerability detection methods, with a focus on source code vulnerability detection based on deep neural networks. Section 3 briefly outlines fundamental technologies related to vulnerability detection in order to better understand our proposed framework. Section 4 describes the CMFVD framework in detail, including aspects such as code preprocessing, slicing methods, and model architecture. Comparative experiments for CMFVD are presented in Section 5. The final section concludes our work by summarizing the performance of CMFVD, identifying limitations, and discussing future directions for development.

## 2. Related Work

Software vulnerabilities refer to software defects that could be maliciously exploited by attackers, leading to unauthorized access, execution of privileged commands, or the acquisition of sensitive information, among other risky operations. To enhance software security, researchers have proposed various vulnerability detection methods. These techniques can be broadly classified into two categories based on whether the corresponding program is executed: static detection and dynamic detection.

### 2.1. Dynamic Methods

Dynamic detection methods are currently the most widely applied approach for vulnerability detection. These methods achieve detection by executing the program in a specific environment and monitoring its runtime behavior. Dynamic detection involves generating program inputs based on relevant strategies, creating random or semi-random test cases within the execution domain, and inputting them into the target program for execution. By analyzing runtime metrics associated with the program, abnormal or erroneous states can be identified to discover unforeseen inputs, guiding code remediation efforts. Among the predominant dynamic detection methods, fuzz testing stands out, with American Fuzzy Lop (AFL) [9], an open-source tool released by Google, as one of the most renowned fuzz testing tools. AFL utilizes coverage-guided seed mutation and test case generation, incorporating numerous improvements and innovations.

Dynamic detection techniques claim a zero false-positive rate, as each occurrence of an abnormal state is observed based on real runtime results. However, this method requires compiling the source code into executable binary files, establishing the corresponding runtime environment, and incurring significant computational overhead, resulting in lower efficiency. Consequently, dynamic detection is typically applicable only for detecting vulnerabilities in binary software when the source code is unavailable. Additionally, due to the inability to precisely locate the code responsible for vulnerabilities, there are limitations in guiding developers to remediate the identified vulnerabilities.

### 2.2. Static Methods

Static detection methods for source code involve analyzing the source code files directly, without actually executing the program, to detect the presence of vulnerabilities. Therefore, static detection methods offer high flexibility, as they do not require the construction of an environment or the compilation of code, eliminating cumbersome processes. However, due to the typically substantial computational effort required to analyze the entire program, static detection methods often need to strike a balance between accuracy and time efficiency.

#### 2.2.1. Rule-Based Methods

Rule-based methods have a relatively straightforward basic concept: predefined rules are established for each type of vulnerability, and these rules are matched by scanning the entire code. Currently, several mature software solutions on the market employ this approach, including FindBugs, Flawfinder, and Checkmarx [9,10,11,12], which directly generate patterns based on source code, as well as Fortify and Coverity [13,14], which use an intermediate code generation approach. However, defining vulnerability rules requires significant human and financial resources, and the quality of rules formulated by experts directly impacts the detection model’s performance. Since these rules are derived based on known vulnerability characteristics, they cannot detect novel vulnerabilities. Furthermore, rule-based detection methods typically exhibit a high false-positive rate, necessitating manual intervention in result verification. Given the high labor costs and low reliability issues, these methods face challenges when applied to the practical detection of vulnerabilities in large-scale software projects.

#### 2.2.2. Deep Learning-Based Methods

In recent years, with the rapid development of deep neural networks and their satisfactory achievements in natural language processing, image processing, and other fields, some scholars have begun to apply these methods to the field of source code vulnerability detection, achieving static detection of vulnerabilities. Data-driven deep learning methods eliminate the need for manual feature extraction and instead autonomously learn and extract features from the samples in the dataset through neural networks.

In the early stages of research, Russell [15] attempted to transfer natural language processing methods to the task of source code vulnerability detection. This method treats source code text as a natural language sequence, converts it into a token sequence through tokenization processing, and then embeds the tokens using Word2Vec [16]. The embedded tokens are input into a CNN for feature extraction. Finally, after processing the extracted features through fully connected layers, a random forest is used for classification. Although the transfer from natural language processing research is simple, experiments have demonstrated the effectiveness of deep learning in the field of source code vulnerabilities, showcasing its immense potential.

Li et al. proposed the VulDeePecker [17] deep learning vulnerability detection framework, which uses a heuristic algorithm to generate code gadgets. Code gadgets are treated as text sequences, tokenized, and then mapped to a high-dimensional vector space using Word2Vec. The core part of the network utilizes Bidirectional Long Short-Term Memory (BiLSTM) neural networks. However, due to limitations imposed by the upstream code parsing software Checkmarx, VulDeePecker only considers source code data flow information and overlooks other information such as control flow and dependencies. 

Representing source code simply as a flat text sequence actually overlooks many important features. By utilizing a graph structure as an intermediate representation for source code, it becomes possible to better illustrate the intricate and nuanced relationships between different statements.

Duan et al. proposed the Vulsniper [18] detection framework. Initially, they parse the source code into a Code Property Graph (CPG) and transform the source code property graph into a 144-dimensional feature vector using a set of custom rules. Subsequently, this vector is input into a BiLSTM for vulnerability detection. While this approach has introduced a graph structure as an intermediate representation for source code, it does not directly use graph structure data for training during the training process. As a result, there is still a certain degree of semantic loss.

Cheng et al. introduced the DeepWukong [19] vulnerability detection framework. This framework transforms source code into an intermediate representation in the form of a PDG and extracts source code features from this sub-graph generated by using code slice. In comparison to the textual representation of source code, the graph structure representation of source code explicitly reveals many features such as data flow relationships and control flow relationships that cannot be expressed through text. 

In addition, beyond directly conducting vulnerability detection on C/C++ source code, similar research and detection methods have been applied to other targets, yielding noteworthy results. These methods continue to offer valuable insights for detecting vulnerabilities in C/C++ source code. Su et al. [20] employed the BERT pre-trained model to detect malicious URL links. Huang et al. [21] converted program binary machine code into grayscale images, utilizing CNN and attention mechanisms for malicious software detection. Lin et al. [22] focused on PHP code, using graph-based intermediate representation to identify web-related vulnerabilities.

## 3. Background

This chapter will introduce graph neural networks, various types of graph-based intermediate representations for source code, and the fundamental concept of code slicing.

### 3.1. Graph-Based Source Code Intermediate Representation

Source code is written and read by people in text form, and although it exists as text, source code should never be treated simply as a text sequence. Programming languages have more complex and strict syntax rules and a broader context compared to natural languages. As the scale of source code increases, analyzing it directly in text form becomes very complex, making it difficult to capture semantic information between distant lines of code. Therefore, in the course of research advancement, graph structures such as Abstract Syntax Tree (AST), Control Flow Graph (CFG), Data Flow Graph (DFG), and others have gradually emerged as intermediate representations of source code.

As a representation independent of specific programming languages, source code graph structures provide a higher-level abstraction at the semantic level and greatly enhance the flexibility of static analysis of source code. They serve as the foundation for current research work.

#### 3.1.1. Control Flow Graph

A Control Flow Graph (CFG) is a directed graph used to illustrate the possible sequence of control flow between different statements in a program, representing all possible program execution paths. The Control Flow Graph provides an intuitive visualization of the program’s execution flow, assisting programmers in better understanding and analyzing the code. In a Control Flow Graph, statements (statement) and predicates (predicate) serve as nodes. Ordinary statements are executed linearly, while branching statements (such as if, else, while, switch) lead to branches in the control flow, with each branch representing a possible control transfer direction.

#### 3.1.2. Data Flow Graph

A Data Flow Graph is a directed graph used to describe the creation, transfer, and usage of relevant variables during program execution. The granularity of a Data Flow Graph is finer than that of a Control Flow Graph, focusing on the specific process of how a particular variable flows and is processed in the program. Directed edges between different nodes represent the paths of usage and transfer for the corresponding variables. A Data Flow Graph provides a clear representation of the dependency relationships in the flow of data, elucidating the input–output relationships between different statements.

#### 3.1.3. Program Dependency Graph

In the development of program slicing techniques and dependency analysis, the Program Dependency Graph (PDG) has been gradually introduced. The PDG uses statements and predicates as nodes, with control dependencies and data dependencies as two types of relationships between different nodes. Specifically, if statement B is executed after statement A, it is referred to as control dependency from statement B to statement A. If the scalar involved in statement B is modified or defined in statement A, it is considered a data dependency from statement B to statement A. Therefore, it can be said that the Program Dependency Graph is constructed based on both the Data Dependency Graph and Control Dependency Graph.

**Figure 1:**
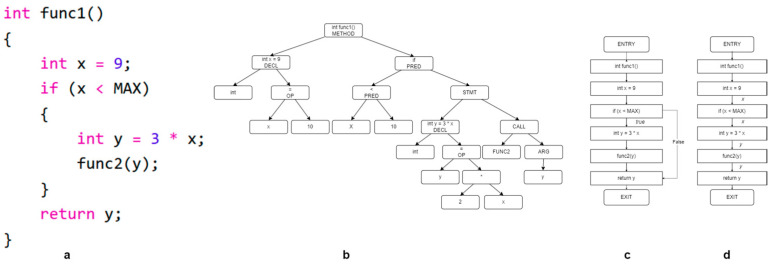
(**a**) Sample code, (**b**) abstract syntax tree, (**c**) control flow graph, (**d**) data flow graph.

**Figure 2:**
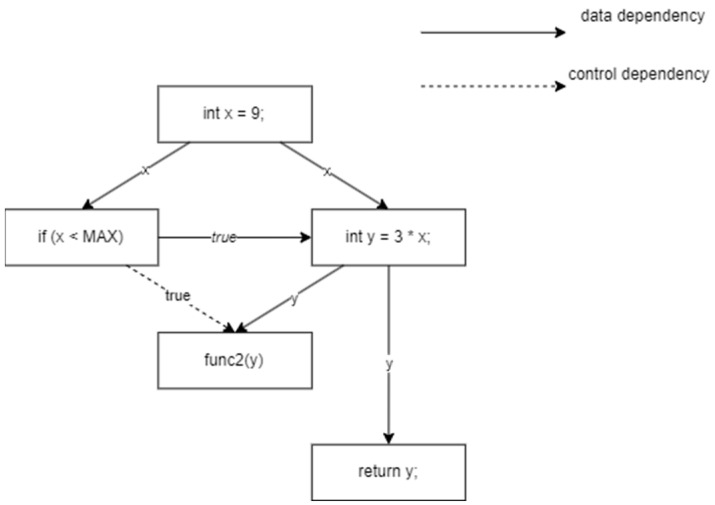
Program dependency graph generated from the example code.

The example code shown in Figure 1 and Figure 2, respectively, illustrates the abstract syntax tree (AST), control flow graph (CFG), data flow graph, and program dependency graph generated from this example code.

### 3.2. Program Slicing Technique

Program slicing, initially proposed by American computer scientist Mark D. Weiser in 1979 [23], is a technique within the domain of the static analysis of the source code. It involves extracting a subset of a specified source code file, a process known as program slicing, resulting in what is referred to as a “slice”. Program slicing starts from a designated code line of interest, referred to as the slice criterion or slice point. By applying program slicing, statements and variables related to the slice point are extracted from the source code, reducing the scope and complexity of the analysis. Program slicing finds wide applications in various commercial development projects to reduce program complexity, assist programmers in locating errors, and alleviate debugging challenges.

According to Weiser’s definition, program slices can be categorized into static slicing and dynamic slicing. Static slicing involves the program slicing process without the actual execution of the program, conducting a comprehensive analysis of program semantics to determine all possible data and control flows. On the other hand, dynamic slicing requires program slicing based on specific contexts, selecting relevant execution paths under certain input conditions to analyze the program’s behavior in specific input scenarios.

In the realm of source code vulnerability detection, researchers often employ forward slicing and backward slicing, both falling under the umbrella of static slicing. The key distinction lies in how they select variables and statements related to the slice point during graph traversal.

- Forward Slicing: Selects all other program parts that may be influenced by the slice criterion.
- Backward Slicing: Selects all other program parts that may influence the slice criterion.

## 4. The Proposed Frame

In this chapter, we will delve into the details of our proposed CMFVD detection framework, a vulnerability detection model based on context slicing and the fusion of multi-dimensional information from graphs and sequences. This includes how to parse source code in textual form into graphs, perform context slicing on the graph, and construct relevant network models, ultimately achieving the prediction of vulnerabilities in the source code.

### 4.1. Overview

**Figure 3:**
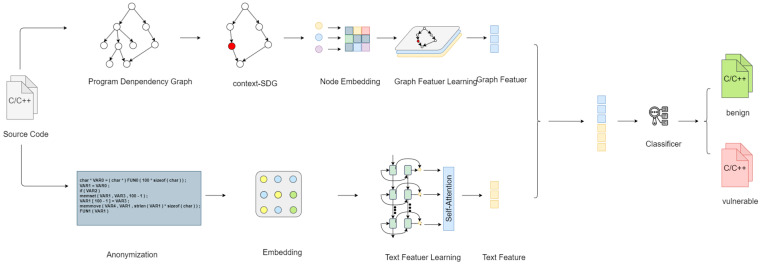
Overall architecture of CMFVD.

The overall design of CMFVD is illustrated in Figure 3. The framework consists of three main parts: source code parsing, feature extraction from sequences and graphs, and feature fusion and classification. Firstly, CMFVD parses source code files to generate corresponding program dependency graphs (PDGs) and utilizes the context slicing method to obtain relevant slices, context-SDG. In context-SDG, a significant amount of semantic and syntactic information related to vulnerabilities is retained. Graph Neural Network and bidirectional gated recurrent unit (BGRU) are employed to extract the graph structure information and textual sequence information from context-SDG, respectively. Finally, these two types of features are fused and fed into the classifier for prediction.

### 4.2. Code Parsing

To achieve slice extraction and convert the processing into a format that the model can receive, source code parsing is required.

#### 4.2.1. Source Code Preprocessing

Firstly, we will remove all commented code from the source code file. Comments, being human-readable natural language hints designed to assist programmers in understanding the logic of the code, are considered to be noise and need to be eliminated in programming languages. Next, we use the Joern [24] tool for parsing the source code, constructing the corresponding code property graph (CPG). Joern is an open-source code analysis tool proposed and implemented by Yamaguchi in the paper [25]. It can transform source code into a CPG graph structure, aiming to assist developers and security researchers in better analyzing and understanding large-scale codebases. CPG integrates various granularities of graph representations, including functions, variables, code lines, control flow, data flow, and related dependencies. By default, Joern outputs two files, nodes.csv and edges.csv, to describe the CPG. The file nodes.csv contains different nodes in the CPG and their related attributes, while edges.csv represents the edges in the CPG through three fields: the start node, end node, and edge type. We comprehensively parse these two CSV files and extract the PDG composed of code lines and the edges representing data dependencies and control dependencies.

#### 4.2.2. Edge Tree

Context slicing is based on the edge tree, and we will start by introducing the concept of the edge tree.

The edge tree is a binary tree whose nodes represent edges in the program dependency graph (PDG). In the process of constructing the edge tree, if there are specific types of connections between edges in the PDG, corresponding nodes in the edge tree (nodes in the edge tree are edges in the PDG) will be connected, forming parent–child relationships.

To facilitate understanding, we will describe this process using formal language.

In the program dependency graph (PDG), an edge, e, can be represented using a triple as follows:

(1) $$e≔(v_{1},v_{2},t)$$

where $v_{1}$ and $v_{2}$ represent the starting and ending nodes, respectively, and t indicates the type of this edge.

In the PDG, for any two edges, $e_{1}$ and $e_{2}$, a relationship relation is defined as follows:

(2) $$relation≔\left(v_{1}=v_{3}\;\cap v_{2}\neq v_{4}\right)\cup \;\left(v_{1}=v_{4}\;\cap v_{2}\neq v_{3}\right)\cup \;\left(v_{2}=v_{4}\;\cap v_{1}\neq v_{3}\right)\cup \;\left(v_{2}=v_{3}\;\cap v_{1}\neq v_{4}\right)$$

When $e_{1}$ and $e_{2}$ satisfy the aforementioned relationship, their corresponding nodes in the edge tree will form a parent–child relationship and be connected. With the above method, we can construct the corresponding edge tree based on the PDG.

#### 4.2.3. Context Slicing

In other research on source code vulnerability detection, the slicing process mostly uses the slicing method proposed by SySeVr [26], which, for clarity, we refer to as BFslicing (backward and forward slicing).

The selection of slice criterion is the starting point for slicing operations. In other works, including SySeVr, four types of slice criterion are typically chosen: sensitive API calls, pointer usage, array access, and arithmetic expressions. Vulnerabilities often occur when these operations are mishandled, such as using dangerous library functions or incorrect array access leading to out-of-bounds errors. Additionally, CMFVD introduces a new type of cut point called ‘extended sensitive functions’. Similar to sensitive API calls, many programmers implement their own memory manipulation functions with custom names that do not easily match the criteria for sensitive API calls. However, there are numerous such functions that may be missed by the model if it cannot fully learn the patterns of vulnerability features. CMFVD defines five types of slice criterion, summarized in Table 1.

BFslicing starts from the slicing point and recursively adds new nodes to the final slicing result using both forward and backward slicing until no new nodes can be added, at which point the slicing process stops.

By observing a large number of slicing instances, we noticed a phenomenon: nodes added at deeper levels often reflect that they are influenced by or are influencing existing nodes based on data dependency relationships. These nodes primarily reflect the semantic flow of data from input to usage. For both vulnerable and non-vulnerable code, the characteristics reflecting the input data flow are often similar. Regardless of the presence of a vulnerability, the received input always arrives at the slicing point along the same data flow path. In other words, the slices obtained for code with and without vulnerabilities contain a lot of redundant content. This low-discriminative information stems from the related input data dependency chain. Such redundant slices are filled with many useless noises, and their low discriminative power not only increases the computational burden during model training but also affects prediction performance.

To address this issue, we believe that the characteristic patterns of vulnerabilities are closely distributed in the vicinity of vulnerable lines, i.e., they exist in the context around vulnerable lines. To overcome the aforementioned problem, we propose an approach called context slicing.

After obtaining the corresponding program dependence graph (PDG), we construct the corresponding edge tree data structure based on the method described in the previous section. Utilizing this edge tree data structure, we perform context slicing. Initially, in the PDG, we choose any edge connected to the slicing point (representing a line of code in the graph) and designate its corresponding node in the edge tree as the starting point. Subsequently, we recursively add nodes directly or indirectly adjacent to the current node to the context slicing result until no new nodes can be added. Afterward, the nodes obtained from the edge tree are mapped back to the corresponding edges in the PDG. We then select the PDG nodes connected to these edges and obtain the final context slice after removing duplicates. Algorithm 1 outlines the process of context slicing.
**Algorithm 1**: Context SlicingInput: Edge Tree ET = {e_1_,e_2_,…,e_n_}. Choose any edge e_k_ in the edge tree that starts from the slicing point.output: context-SDG   result = {e_k_}   queue = {e_k_}   while queue ! = ∅ do      for e_i_ in queue do         for e_j_ in get_direct_or_indirect_neighbors(e_k_) do            if e_j_ not in result do               result.add(e_j_)            end if         end for      end for   end for    context_SDG = {}   for e_i_ in result do      for node in get_ successors_and_ predecessors(e_i_) do         if node not in context_SDG do            context_SDG.add(node)         end if      end for   end for    return context_SDG

Next, we will illustrate the specific processes of BFslicing and context slicing using a practical example, as well as highlight the differences between these two methods.

**Figure 4:**
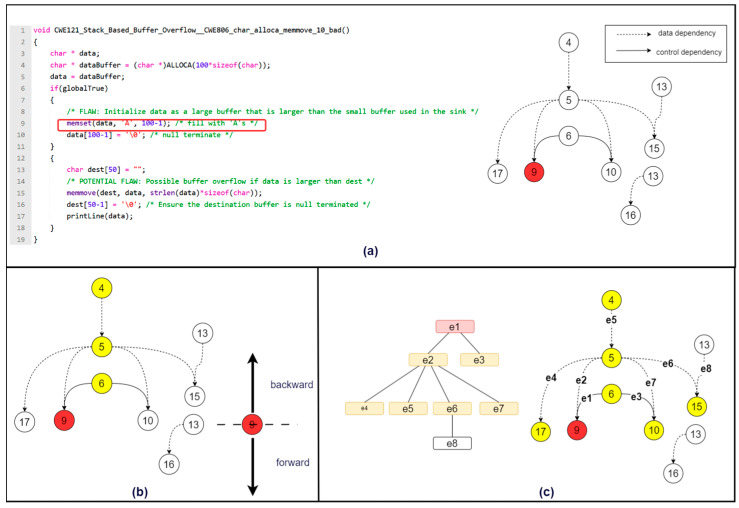
(**a**) The example code and its corresponding PDG, with the line highlighted in red as a slice criterion, (**b**) illustration of BFslicing on the example code, (**c**) illustration of context slicing on the example code.

Figure 4a shows the sample code along with its corresponding program dependency graph (PDG), and we choose the ninth line of code as the slicing point.

In BFslicing, we start from the slicing point 9 and recursively call forward and backward slicing to add new nodes. At node 9, forward slicing cannot add any new nodes, while backward slicing appends nodes 5 and 6. Subsequently, we apply backward slicing again at nodes 5 and 6. According to the backward slicing rules, node 6 cannot append any new nodes. However, through backward slicing, we can append node 4 after node 5. Then, when using backward slicing again at node 4, no new nodes can be appended, concluding the entire slicing process. The final results of forward and backward slicing are [4,5,6,9]. Figure 4b illustrates this process.

For context slicing, we first construct the corresponding edge tree based on the program dependence graph (PDG). We select the edge $e_{1}$ directly connected to the slicing point as the root of the edge tree. Based on the definition of the relation described earlier, we find that both $e_{2}$ and $e_{3}$ satisfy the relation with $e_{1}$. In the edge tree, the nodes representing these two edges become children of $e_{1}$. Similarly, $\left[e_{4},e_{5},e_{6},e_{7}\right]$ become children of $e_{2}$, and $e_{8}$ becomes a child of $e_{6}$. Next, we perform context slicing on the edge tree. Following the algorithm for context slicing, we choose nodes in the edge tree that have a direct or indirect relation with $e_{1}$, resulting in $\left[e_{2},e_{3},e_{4},e_{5},e_{6},e_{7}\right]$. Finally, we need to map the edge tree slicing results back to the PDG. The specific method involves selecting the start and end nodes of the edges chosen during the edge tree slicing process and adding them to the final slicing results. For example, for edge $e_{1}$, we add the nodes [6,9] to the final slicing results. For edge $e_{2}$, we should add the nodes [5,9], but since 9 already exists, we do not duplicate it. Summing up and removing duplicates yields the slicing result [4,5,6,9,10,15,17]. Figure 4c illustrates the process of context slicing on the example code.

Through the above example, it is evident that, compared to BFslicing, context slicing selects more nodes [10,15,17]. The lines of code corresponding to these three nodes involve write operations on the variable “data”. For a pointer pointing to an array, understanding write operations on the corresponding memory space is crucial, as memory errors often result from illegal write operations, leading to vulnerabilities. However, BFslicing does not include any information related to write operations on the data pointer.

The reason BFslicing misses these lines of code is clear: nodes newly added through forward slicing will only go through forward slicing, and the same applies to nodes added through backward slicing. These lines of code are located after the ninth line of code (line 9), but PDG node 9 cannot obtain any new nodes through backward slicing. As a result, crucial information is lost. This precisely reveals the shortcomings of forward and backward slicing methods: if a node cannot add new nodes through forward or backward slicing in the slicing process, it will cause the slicing chain to break. In such cases, even if certain nodes are significant, they may be lost because their predecessor nodes or successor nodes were not appended to the previous round of slicing results.

In contrast, context slicing shifts the primary focus of slicing from nodes to edges. It completes the slicing process within the edge tree and then maps the sliced results of edges to nodes in PDG to obtain the final slicing results, overcoming this limitation. This approach addresses the problems faced by traditional methods and enhances accuracy and reliability. Moreover, it is evident that BFslicing provides a “long and slim” slicing result, as it traces the initial input and final usage of variables through data dependency relationships. Regardless of whether the related variables lead to vulnerabilities due to erroneous operations, their input data dependency chains always remain similar. Context slicing provides a “short and wide” slicing result, concentrating on including lines of code closely related to vulnerability patterns as vulnerability context information.

Moreover, through the example code, we can intuitively appreciate that, in cases where the software is not particularly deep or complex, context slicing often complements forward and backward slicing. In the slicing results demonstrated in this example, the lines of code obtained through BFslicing are all present in the results of context slicing.

After obtaining the context slices, we will save the corresponding lines of code in the source file based on the selected nodes for later use in graph node embedding and sequence network input. These lines of code are stored in text form, similar to natural language processing tasks, and may require preprocessing when dealing with these strings.

First, it is necessary to remove the comments from the code lines since comments are hints for programmers to understand the logic and are considered human-readable natural language. Next, the code strings undergo tokenization. Specifically, tokenization transforms the code string into a sequence of tokens composed of the smallest syntactic units, including variable names, operators, constants, and keywords. For example, “char dataBuffer = (char)ALLOCA(100 * sizeof(char))” would be converted to the sequence “[char, dataBuffer, =, (, char, ALLOCA, (, *, sizeof, (, char,),)]”.

**Figure 5:**
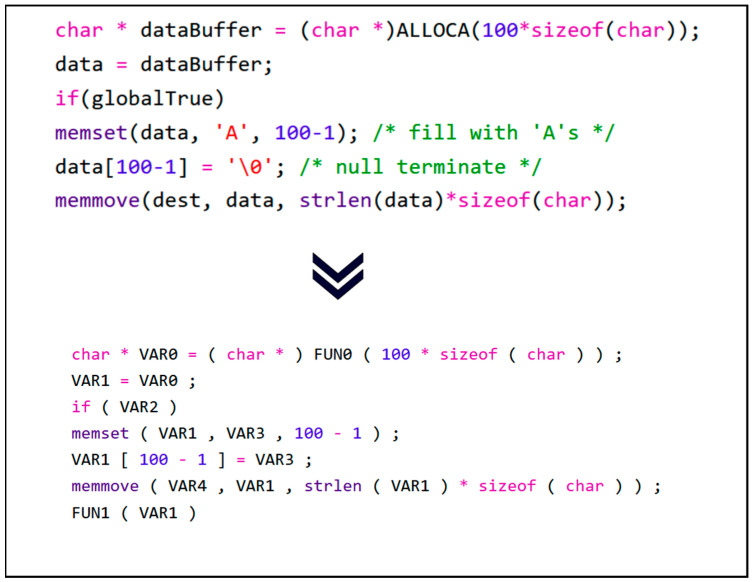
Anonymization process of the sample code.

Following that, user-defined variable names and function names in the source code are mapped to a unified and standardized style. Source code contains a multitude of elements composed of user-defined function names, variable names, and other identifiers. Different coding styles and personalized variable naming practices among programmers result in logically and functionally similar but significantly different code at the textual feature level. The presence of different coding styles and personalized variable naming introduces noise. This makes it challenging for neural networks to establish semantic correlations between logically similar codes, thereby affecting vulnerability feature model learning and ultimately reducing the detection model’s performance. For each token sequence, user-defined variable names and function names are replaced with VAR0, VAR1, …, VARn and FUN0, FUN1, …, FUNm, respectively, based on their appearance order. Additionally, string constants appearing in the source code are uniformly replaced with an empty string. Through anonymization, source code with similar semantic and structural characteristics will have a unified naming style, ensuring that subsequent models better understand code feature patterns and greatly reducing the size of the vocabulary. Figure 5 illustrates the anonymization process described above.

### 4.3. Feature Extraction of Graph and Sequence

#### 4.3.1. Extraction Global Semantic Information Based on Graph

For the nodes in the graph, we use the Doc2Vec [27] model to encode the text of each code line corresponding to each node, obtaining feature embeddings for each node. Doc2Vec is an extension of the Word2Vec model that extends embeddings from the word level to the entire sequence level, representing embeddings for entire documents.

**Figure 6:**
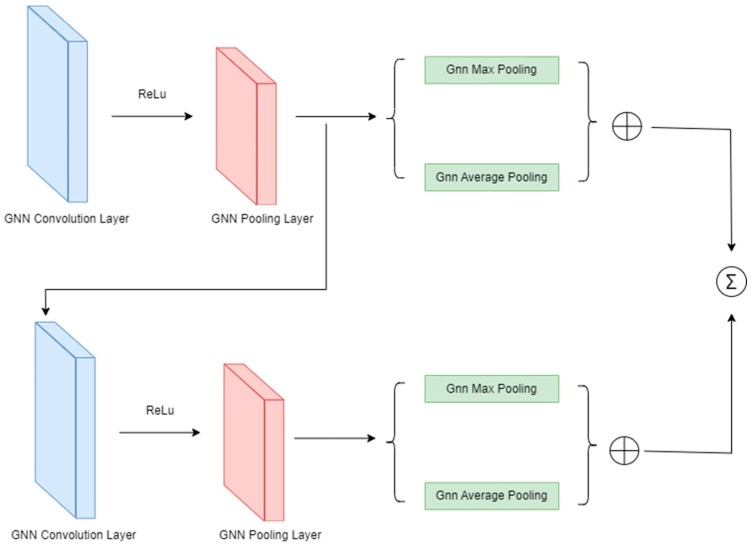
Network architecture for extracting graph structural features.

The structure of the feature extraction network in this framework is illustrated in Figure 6. Overall, the main part of the model consists of stacked layers of GCN convolution-pooling blocks. Each convolution-pooling block is composed of a graph convolution module and a pooling module. The convolutional layer in the block uses ReLU [28] as its non-linear activation function to enhance the expressive power of the network. The convolutional layer guides the propagation, aggregation, and updating of features among neighboring nodes. After convolution, a vector representation of the entire graph can be obtained.

For the pooling layer, the Top-k pooling method is chosen, meaning that only the top-k most important nodes are retained during each pooling operation, and the other nodes are discarded. The chosen pooling parameter is set to 0.8. Specifically, the framework simultaneously employs both max pooling and average pooling methods, concatenating their outputs. This allows each node in the graph to comprehensively represent information about the entire graph. This pooling operation effectively reduces the number of nodes in the graph, mitigating computational complexity, retaining crucial information, reducing model training parameters, and preventing overfitting. For the graph readout layer, a jk-style graph [29,30] readout layer is used, preserving the output of the intermediate pooling processes and combining them through concatenation to form the final vector representation.

#### 4.3.2. Extraction Local Syntax Information Based on Sequence

Through the previous methods, we currently have token sequences corresponding to cs-SDG after anonymization. Next, we will discuss how to extract local syntactic information from this text sequence.

Each element in the anonymized token sequence is still in text form and cannot be directly input into the sequence neural network. Therefore, we use Word2Vec [17] to embed text tokens into a vector space, transforming them into vectorized data that can be processed by the network model. Word2Vec is trained based on the co-occurrence information between tokens, mapping each token to a continuous high-dimensional vector. Since the vector space is trained based on the contextual relationships between different tokens, the distance between tokens in this vector space can reflect their semantic associations, meaning tokens with similar semantics have closer Euclidean distances.

This framework employs the bidirectional gated recurrent unit (BGRU) [31] model to extract syntactic information from the sliced source code text. BGRU is an improved version of the gated recurrent unit (GRU) [32] that stacks two GRU networks in different directions. Compared to Long Short-Term Memory (LSTM) [33], which is an improved version of the LSTM, GRU has a more concise structure and fewer parameters, exhibiting better performance and faster training speed on tasks with lower complexity. In contrast to LSTM, GRU simplifies the design by reducing the forget gate, input gate, and output gate to two key units: the reset gate and the update gate.

In essence, GRU is designed to control the forgetting of less important previous state information through the reset gate at each time step and to control the appending of new hidden states relevant to the current time step through the update gate.

For programming languages, even local syntax information contains complex details compared to traditional natural languages. Therefore, after BGRU, we introduce a self-attention module. By incorporating the self-attention module, the model can better understand information at different positions in the sequence, focus on important time steps, reduce attention to irrelevant tokens, and enhance its ability to understand contextual features. This significantly improves the model’s ability to handle long-distance dependencies, thereby enhancing performance, expressiveness, and generalization capability.

#### 4.3.3. Feature Fusion and Classification

Through the two aforementioned sub-modules, we obtain feature vectors representing different dimensions of source code: graph structure features and recurrent network sequence features. In this paper, we adopt a simple concatenation method to merge these two types of features and obtain the final feature vector. Although the concatenation method is straightforward, it has proven to be effective. The resulting feature vector from concatenation includes global semantic information such as data and control dependencies, as well as local syntactic information such as variable types and branching conditions. Subsequently, we input the fused feature vector into a Multilayer Perceptron (MLP) for prediction. The MLP has a simple yet powerful structure, consisting of only two fully connected layers; however, it exhibits excellent non-linear modeling capabilities and performs well when handling complex multi-dimensional data features. Finally, to predict whether a given code contains a vulnerability, softmax is used as an activation function before the MLP output to transform it into a probability range for prediction.

(3) $$p=softmax(mlp([x_{graph},x_{attn\_sequence}]))$$

Here, p={(x,y)|x+y=1, 0<x<1, 0<y<1}, where x and y represent the probabilities of the existence and non-existence of vulnerabilities, respectively. We choose the probability value with the higher likelihood as the prediction result for this instance.

## 5. Experimental Results and Analysis

### 5.1. Dataset

CWE (Common Weakness Enumeration) [34] is a database maintained by MITRE Corporation, designed to categorize vulnerabilities and assist global researchers in understanding the causes, characteristic patterns, potential impacts, and remediation methods of vulnerabilities. CWE organizes different types of vulnerabilities by assigning a unique CWE-ID to each vulnerability category, presented in a tree-like structure with some degree of inheritance in parent–child relationships.

To assess the detection performance of our framework, we selected five CWE vulnerability types related to memory from CWE 2022 Top 25 [35]: CWE-121, CWE-122, CWE-125, CWE-124, and CWE-476. Memory-related vulnerabilities are the most common types in C/C++, so we have chosen the aforementioned five CWE types, which are related to stack overflow, heap overflow, out-of-bounds access, and pointer issues. Essentially, they all pertain to memory vulnerabilities. The specific information about these five vulnerability types is shown in Table 2.

SARD (Software Assurance Reference Data Set) [36] maintains a test suite called Juliet C/C++. The Juliet Test Suite for C/C++ [37] consists of 64,099 C/C++ language test code samples, covering 118 different types of CWE vulnerabilities. These vulnerabilities include common C/C++ issues such as buffer overflow, integer overflow, and null pointer dereference. For each test sample, SARD marks the lines where vulnerabilities exist and provides the corrected code. The correction involves minor modifications to the source code, with differences limited to aspects like branch conditions, variable declarations, and function calls, ensuring the generation of a large and well-balanced dataset. We selected the aforementioned five types of vulnerabilities to evaluate the detection performance of CMFVD.

To evaluate the performance of CMFVD across different CWE types, experiments were conducted separately on the five selected CWE categories. Each CWE type was randomly divided into training, validation, and test sets in an 8:1:1 ratio.

### 5.2. Metrics

To quantify the detection performance of CMFVD, we utilized classical classification evaluation metrics, including accuracy (ACC), precision (P), recall (R), and False Positive Rate (FPR). Additionally, to comprehensively assess the trade-off between accuracy and precision, the F1-score was introduced. To calculate these evaluation metrics, the confusion matrix was formed based on the model’s predictions. The confusion matrix comprises four components: TP (True Positive), FP (False Positive), TN (True Negative), and FN (False Negative). Each row in the matrix represents the model’s predictions, while each column represents the actual conditions of the corresponding samples.

The accuracy is a comprehensive metric that represents the proportion of correctly predicted samples to the total number of samples. It evaluates the model’s predictive performance for both positive and negative samples.

Precision is the proportion of correctly predicted positive samples among all predicted positive samples. It reflects the model’s ability to predict positive samples accurately.

The False Positive Rate (FPR) measures the proportion of incorrectly predicted negative samples among all predicted positive samples. It assesses the rate of false alarms in positive predictions.

Recall, also known as the true positive rate, is the proportion of correctly predicted positive samples among all actual positive samples. It reflects the model’s ability to capture true positive samples.

The F1-score is a harmonic mean of precision and recall, providing a balanced assessment of the model’s overall performance. It considers both precision and recall, offering a comprehensive evaluation.

### 5.3. Experiment Environment

The experimental setup for this research involved a workstation with 64 GB of RAM, an Intel Xeon Silver 4210R processor, and an NVIDIA GeForce RTX 4090 graphics card running Ubuntu 22.10. For a detailed configuration list, please refer to Table 3.

We employed a batch training approach with each batch containing 64 samples. The feature vector dimension for each token was set to 64, the dropout rate was configured at 0.5, and the graph pooling rate was set to 0.8. The maximum number of training epochs was set to 200, and we used the ADAM optimizer for parameter optimization. The activation function selected was RELU, and the learning rate was set to 0.001.

### 5.4. Result and Analysis

We conducted two sets of experiments to validate the effectiveness of the slicing method proposed in this paper and the model construction.

Experiment 1: Is context slicing effective?

**Figure 7:**
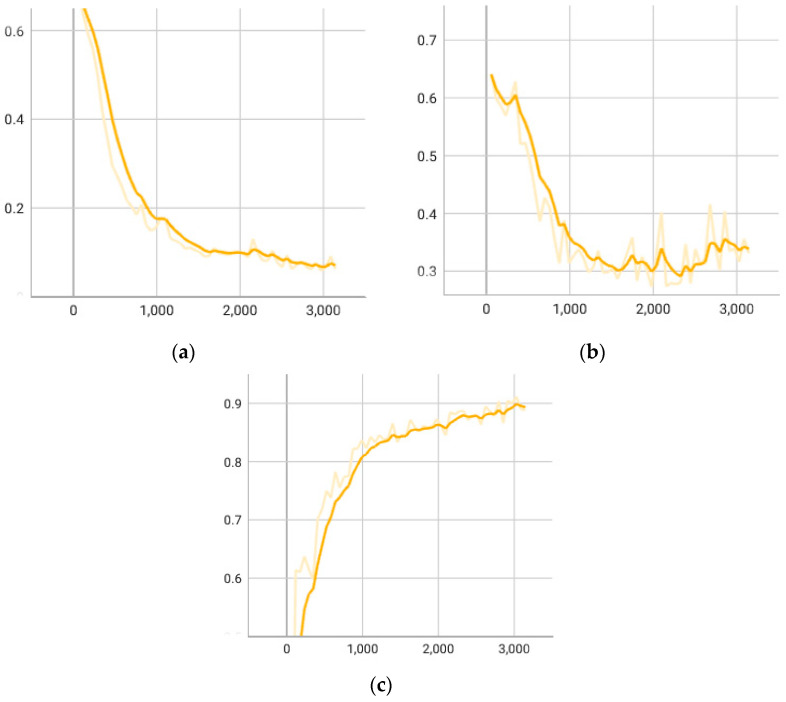
CMFVD metrics: (**a**) CMFVD train loss; (**b**) CMFVD validation loss; (**c**) CMFVD validation F1-score.

To validate the effectiveness of the proposed context slicing method in this paper, we conducted comparative experiments on the proposed model architecture, focusing on the five mentioned CWE types. One experiment utilized the traditional slicing method, combining forward and backward slicing, while the other experiment employed the proposed context slicing method. Table 4 presents the experimental results. The changes in loss and F1-score during the training and validation stages are illustrated in Figure 7. It can be observed that the model using the context slicing method outperformed the traditional slicing method in each evaluation metric. Specifically, for buffer overflow-related vulnerabilities (such as CWE-121, CWE-122, CWE-124), the model using context slicing performed well, with an average F1-score exceeding 0.95. However, in CWE-476, there was a performance degradation issue for both context slicing and traditional slicing methods, with a noticeable decrease in precision and F1-score around 0.83. This is mainly because CWE-476 belongs to the null pointer dereference vulnerability type, which heavily relies on the program’s dynamic environment. For instance, common dynamic memory allocation functions like malloc may return a pointer that could be null based on runtime conditions, making it challenging to determine the specific return value during static analysis. Additionally, null pointer dereference vulnerabilities often involve a series of pointer operations that are difficult to accurately trace during static analysis.

Experiment 2: Does the introduction of sequence information improve the performance of the model?

To validate the effectiveness of introducing text sequence information corresponding to slices, this paper chose the DeepWukong network architecture for experiments. The DeepWukong model utilizes only the slicing’s graph structure information for training, meaning it learns vulnerability feature patterns through global syntax information. We independently reproduced the DeepWukong model architecture based on relevant papers and conducted experiments on the same context slicing dataset. The experimental results are detailed in Table 5. We observe that CMFVD performs better than DeepWukong in all five datasets, with F1-scores exceeding 0.91 for all CWE types except CWE-476. Coincidentally, just like the comparative experiment with slicing, both models encountered issues in terms of performance degradation for CWE-476. Firstly, we believe that insufficient training sample quantity is a limiting factor. As shown in Table 2, among all CWE types, there are less than 1000 slices for CWE-476 alone, whereas other vulnerability types have at least three times more samples than CWE-476. This lack of sample quantity restricts the model’s performance. Secondly, compared to other vulnerability types, null pointer dereference has more complex data flow and control flow information. When a pointer is freed but not set to null, it may be dereferenced again far away. This enlarges the context window and makes the content more intricate, making it difficult to capture vulnerability patterns through simple methods. The scarcity of data further amplifies this drawback. Nevertheless, this still indicates that introducing local syntax information in the sequence can indeed complement global semantic information from the graph structure and contribute to improving detection performance.

Experiment 3: Can CMFVD detect vulnerabilities in real code?

The SARD dataset used for training is semi-synthetic, consisting of generated code and real source code donated by organizations. It is undeniable that there are certain differences between the semi-synthetic code and real samples. The code in the SARD dataset is relatively simple and mainly used to directly demonstrate vulnerability patterns. However, in the real world, code often produces certain forms of vulnerabilities due to negligence in business logic. Therefore, triggering conditions for vulnerability patterns in real code are more complex. Nevertheless, we firmly believe that CMFVD can learn the vulnerability characteristics of real samples in the SARD dataset. To evaluate CMFVD’s ability to detect vulnerabilities in real code, we applied the CMFVD framework for vulnerability detection on FFmpeg’s code. Table 6 shows some of the vulnerabilities discovered by CMFVD in FFmpeg.

**Figure 8:**
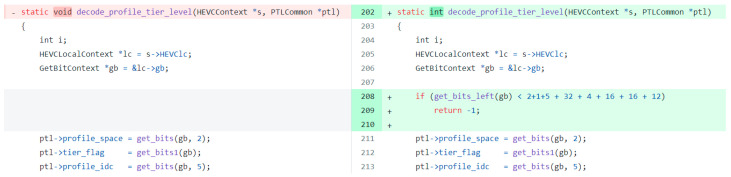
Code details of the commit.

In addition, CMFVD has also discovered some vulnerabilities that have not been reported in CVE. Figure 8 shows the detail of one of the vulnerabilities and its first-six commit id is 0d0d24. Due to the lack of boundary checks for *gb*, there is a potential risk of buffer overflow. In the criterion of utilizing pointer usage and extending sensitive function calls, CMFVD successfully identified the *gd* variable and detected its insufficient boundary check during feature extraction in neural networks through context slicing. Consequently, this vulnerability was detected.

## 6. Conclusions

To enhance model detection performance and address limitations in existing research methods, we propose CMFVD, a vulnerability detection framework that integrates graph and sequence information using the context slicing method. The context slicing method addresses issues present in commonly used slicing methods BFslicing (combining forward and backward slicing) in current source code vulnerability detection research:

- High Similarity in Slices: Slices obtained for both vulnerable and non-vulnerable code exhibit high similarity, leading to low distinguishability.
- Missing Important Statements: Due to the limitations of forward and backward slicing, crucial statements related to vulnerability patterns may be overlooked in cases of “broken chains”.

To address these issues, we introduce the context slicing method and recognize the significance of code text sequence information, providing local syntax information for the model to learn vulnerability patterns. Consequently, we designed the CMFVD framework, a combination of a sequence and graph structure network model. In summary, CMFVD is a vulnerability detection framework that utilizes the context slicing method, combining graph and sequence information. Experimental results on the SARD dataset validate the effectiveness of these improvements, achieving an average F1-score exceeding 0.90. The framework exhibits potential application value building upon existing state-of-the-art models.

However, CMFVD still has room for improvement: at the current level, it cannot precisely locate vulnerabilities, and further efforts are needed to narrow down the detection granularity for precise localization. Additionally, the main experiments focus solely on binary classification tasks. Future work will concentrate on exploring more fine-grained detection methods and constructing multiclass networks.

## Figures and Tables

**Table 1:** Information for different criterion types.

Criterion	Description	Example
Sensitive API	The invoked function name is included in the pre-defined list of dangerous APIs	strcpy(dest, src);
Pointer usage	Using the dereference symbol ‘*’	int * a = (int *)malloc(sizeof(int) * 5);
Array access	Accessing array elements using ‘[]’	array [2] = ‘q’;
Arithmetic expression	Using arithmetic operators such as ‘+’, ‘−’, ‘*’, and ‘/’.	index = index − offset;
Extended sensitive functions	The function called with a parameter of pointer type, which is not included in the list of dangerous API calls.	transform(pointerA, pointerB);

**Table 2:** Information for different CWE types.

CWE-ID	Description	Slices
CWE-121	Stack-based Buffer Overflow	8367
CWE-122	Heap-based Buffer Overflow	11,743
CWE-124	Out-of-bounds Read	4564
CWE-127	Buffer Underwrite (‘Buffer Underflow’)	3535
CWE-476	NULL Pointer Dereference	945

**Table 3:** Experimental environment configuration.

Type	Config
CPU	Intel Xeon Silver 4210R (20) @ 3.200 GHz
GPU	NVIDIA GeForce RTX 4090 CUDA 12.0
RAM	64 G
OS	Ubuntu 22.10 x86_64
Environment	Python 3.10, Pytorch 1.13.1

**Table 4:** Different slicing methods’ results comparison on different dataset (CS represents context slice, and BF represents forward and backward slice).

Dataset	Slicing Method	Accuracy	Precision	Fpr	Recall	F1
CWE121	CS	0.952	0.940	0.054	0.959	0.950
	BF	0.896	0.820	0.097	0.882	0.850
CWE122	CS	0.985	0.986	0.013	0.982	0.984
	BF	0.898	0.840	0.087	0.870	0.854
CWE124	CS	0.952	0.935	0.033	0.980	0.946
	BF	0.915	0.815	0.110	0.964	0.883
CWE127	CS	0.924	0.880	0.100	0.956	0.916
	BF	0.903	0.863	0.110	0.921	0.891
CWE476	CS	0.936	0.792	0.067	0.950	0.836
	BF	0.920	0.739	0.086	0.940	0.829

**Table 5:** Different methods’ results comparison on different datasets.

Dataset	Method	Accuracy	Precision	fpr	Recall	F1
CWE121	CMFVD	0.952	0.940	0.054	0.959	0.950
	DeepWukong	0.912	0.873	0.121	0.949	0.910
CWE122	CMFVD	0.985	0.986	0.013	0.982	0.984
	DeepWukong	0.906	0.855	0.156	0.971	0.910
CWE124	CMFVD	0.952	0.935	0.033	0.980	0.946
	DeepWukong	0.921	0.892	0.085	0.926	0.910
CWE127	CMFVD	0.924	0.880	0.100	0.956	0.916
	DeepWukong	0.906	0.901	0.075	0.883	0.892
CWE476	CMFVD	0.936	0.792	0.067	0.950	0.836
	DeepWukong	0.904	0.762	0.068	0.800	0.780

**Table 6:** A part of vulnerabilities detected by CMFVD in real code.

CVE ID	File	Id (First 6 Digits)
CVE-2020-20891	/libavfilter/vf_gblur.c	64a805
CVE-2021-33815	/libavcodec/exr.c	26d3c8
CVE-2017-9990	/libavcodec/xpmdec.c	cb2439

## Data Availability

Data are contained within the article.
